# Revealing New Patterns in Colorectal Cancer Screening with a Focus on a Younger Patient Population

**DOI:** 10.3390/cancers17101686

**Published:** 2025-05-16

**Authors:** Lynette Sequeira, Dhananjay Vaidya, Jianqiao Ma, Aarav Bansal, Shanshan Huang, Ashish Nimgaonkar, Ekta Gupta

**Affiliations:** 1 Department of Internal Medicine 1, School of Medicine, Johns Hopkins University, Baltimore, MD 21218, USAjma75@jh.edu (J.M.); 2 Department of Gastroenterology and Hepatology, School of Medicine, Johns Hopkins University, Baltimore, MD 21218, USA; abansal31@students.gilman.edu (A.B.); shuang52@jhmi.edu (S.H.); animgao1@jh.edu (A.N.); egupta3@jhmi.edu (E.G.)

**Keywords:** colorectal cancer, cancer screening, healthcare disparities

## Abstract

Colorectal cancer (CRC) continues to impart a significant mortality burden in the United States, with a growing number of cases affecting younger individuals. In this study, we set out to characterize predictors of missed colorectal cancer screening in a general and age-stratified population. **Methods**: We analyzed a patient population of over 85,000 patients who presented to a large outpatient network in the Baltimore, Maryland area and were due for CRC screening. We analyzed different characteristics, including race, occupation, relationship status, tobacco smoking status, and body mass index, of patients up to date and overdue on their CRC screening. The majority (over 99%) of our patient population was insured. We performed this analysis on the patient population as a whole and as an age-stratified patient population. **Results**: In our overall patient population, all of the aforementioned characteristics were significantly different between patients up to date and those overdue on CRC screening. Races with the highest up-to-date CRC screening proportion were Pacific Islanders, East Asian, and White patients, while Asian Indian patients had the lowest up-to-date percentage. Non-employed patients (including patients with disabilities and students), single patients, and current or past tobacco smokers were all found to have significantly lower percentages of up-to-date patients as compared to other groups within these categories. BMI was significantly lower in up-to-date patients. In our age-stratified analysis, younger patients had a significantly lower percentage of up-to-date patients. Notably, younger patients had a significantly higher proportion of patients electing for noninvasive screening modalities. **Conclusions**: These disparities in CRC screening warrant targeted interventions to minimize future risk of heightened mortality in certain patient populations.

## Simple Summary

With the growing disease burden of colorectal cancer (CRC) screening in both the general and younger patient populations, it is important to identify disparities in screening that may be amenable to targeted intervention. In our study, we analyzed both traditional elements known to contribute to overall health-related disparities (e.g., race, occupation) as well as nontraditional elements (e.g., relationship status, smoking status, body mass index), all of which we demonstrated to have some effect on successful CRC screening completion in both general and age-stratified patient populations.

## 1. Introduction

### 1.1. Background

Colorectal cancer (CRC) is an increasingly common disease in the United States, with a lifetime incidence in average-risk patients of approximately four percent. As estimated by the National Cancer Institute’s Surveillance, Epidemiology, and End Results (SEER) program, the estimated new CRC cases in the United States of America (US) in 2024 will be 152,810 and will account for 7.6 percent of new cancers [1]. It also imposes a significant mortality burden, with an estimated 53,010 deaths estimated in 2024 alone, and consistently contributes as one of the top three highest cancer-related incidence and mortality rates in the past 5 years in the US [1].

Given this high incidence and mortality burden of CRC, the United States Preventative Services Task Force (USPSTF) recommends screening all average-risk individuals aged 50 to 75 years (Grade A recommendation) and average-risk individuals aged 45 to 49 years (Grade B recommendation) [2]. Average-risk individuals are defined as those with no prior diagnosis of colorectal cancer, adenomatous polyps, or inflammatory bowel disease and those with no personal diagnosis or family history of known genetic disorders that predispose them to a high lifetime risk of colorectal cancer (such as Lynch syndrome or familial adenomatous polyposis). Screening modalities include the high-sensitivity guaiac fecal occult blood test (HSgFOBT) or the fecal immunochemical test (FIT) every year, stool DNA-FIT every 1 to 3 years, computed tomography colonography every 5 years, flexible sigmoidoscopy every 5 years, flexible sigmoidoscopy every 10 years with annual FIT, or colonoscopy screening every 10 years. Age-appropriate, on-time screening has been shown to significantly reduce colorectal cancer risk and mortality [3]. Nevertheless, prior investigation has established that there exist significant disparities in the completion of on-time CRC screening, likely resulting in disproportionate mortality burdens among certain populations [4,5,6,7,8]. In our study, we set out to comprehensively evaluate characteristics of both a general and age-stratified population to evaluate for disparities that may contribute to an inability to complete on-time CRC screening, especially in younger patients.

Specifically, we evaluated patients’ age, gender identity, race/ethnicity, occupation, relationship status, tobacco smoking status, body mass index (BMI; noted in kilograms per meter squared or kg/m^2^), and type of screening modality used. Prior research has established that patients’ health insurance status plays one of the most important roles in on-time colorectal cancer screening completion [9]. We chose to analyze factors outside of insurance status to evaluate what other, if any, disparities exist between up-to-date patients and those overdue on CRC screening. For instance, colonoscopy—the gold standard of CRC screening—typically requires patients to have someone to transport them home due to the effects of anesthesia, and this person may often be a significant other. Thus, we postulated that single patients may have a more difficult time in completing on-time CRC screening due to difficulty finding transportation after colonoscopy. Additionally, tobacco smoking and obesity are both known risk factors for the development of CRC, making current or prior tobacco smokers and obese patients extremely important patient populations to receive on-time CRC screening [10]. Therefore, we set out to identify whether these characteristics influenced on-time CRC screening as well.

### 1.2. Study Objectives

The primary aim of this study was to assess and compare characteristics of patients (including the type of CRC screening modality chosen) who completed and were unable to complete age-appropriate colorectal cancer screening at a large tertiary care center. Our secondary aim was to identify the characteristics of younger patients that influence successful CRC screening completion.

## 2. Materials and Methods

### 2.1. Study Design

This was a retrospective analysis utilizing de-identified Electronic Health Records (EHR) from Johns Hopkins Medical Institutions’ (JHMI) primary care centers. This is a network of 106 primary care centers in and around the Baltimore area in Maryland, United States. This study was approved by the JHMI Institutional Review Board.

The study population included patients aged 50–75 (inclusive) who were enrolled in JHMI Primary Care between the years of 2016 and 2024. All patients were included only once (i.e., if a patient was screened multiple times, they were included only once in the analysis). The initiation of data collection in 2016 preceded the change in the recommended age of initiation of CRC screening from 50 to 45 years, which occurred in 2021. Therefore, we set our lower age limit to 50 years old.

All included patients were required to have established care (i.e., at least one primary care visit) at one JHMI primary care office at any point during the studied time frame (1 January 2016 to 31 December 2024). Patients were required to have attended their primary care visits. Patients who presented for urgent care visits or post-hospital/Emergency Department follow up visits were excluded from analysis. Due to quality care requirements at our institution, all patients were required to have their CRC screening status documented during their primary care visit. Physicians are not able to sign visits unless this is documented, which ensures the accuracy and reliability of CRC screening status.

### 2.2. Study Outcomes

The primary study outcomes were patients’ age, gender identity, race/ethnicity, occupation, relationship status, tobacco smoking status, body mass index (BMI; noted in kilograms per meter squared or kg/m^2^), and type of screening modality used in two groups of patients: those who successfully completed CRC on time and those who did not. Adherence to age-appropriate CRC screening was determined and defined using the aforementioned USPSTF guidelines. Patients’ races were self-reported and entered into the EHR. The race of 0.01 percent of our patient population was unknown. Occupation, relationship status, and tobacco smoking status were also self-reported by patients. These variables were collected at the time of primary care visits. We were able to capture the relationship and tobacco smoking status of all patients and were able to capture the occupational status for 99.7 percent of our patients. Finally, BMI was calculated using available weight and height data in the EHR at the time of CRC screening completion.

Our secondary outcome was to evaluate if these characteristics differ in younger versus older patients, given the recent change in CRC screening recommendations to encompass a younger patient population. Therefore, we performed an analysis of these study outcomes on three groups of patients: (1) all patients aged 50 to 75 years, (2) patients aged 50 to 55 years, and (3) patients aged 56 to 75 years. The first patient population provides insight into the overall characteristics of patients who successfully completed and were unable to complete CRC screening. The initiation of our data collection preceded the change in recommended CRC screening guidelines to include patients ages 45 to 50 years old. Therefore, we evaluated the younger patients in our patient population (aged 50 to 55 years) to serve as a surrogate to estimate and predict the characteristics of patients that influence successful CRC screening completion at a younger age.

### 2.3. Statistical Analysis

Statistical analysis for all categorical variables (race, occupation status, relationship status, and tobacco smoking status) was performed using a chi-squared analysis, and statistical analysis for continuous variables (BMI) was performed using a two-tailed *t*-test.

## 3. Results

### 3.1. Analysis of All Patients

Our primary aim was to evaluate differences between patients who successfully underwent CRC screening and those who did not. The primary outcomes that we investigated were race/ethnicity, occupation, relationship status, tobacco smoking status, BMI (noted in kilograms per meter squared or kg/m^2^), and type of screening modality used.

With regard to race, we found that Pacific Islanders had the highest proportion of patients with up-to-date screening (85.2%), followed by East Asian (82.3%), White (79.1%), American Indian/Alaskan Native (AI/AN; 78.7%), Black/African American (77.1%), and Asian Indian (74.9%). Using Pearson’s chi-squared test, all these proportions were statistically significantly different (*p* < 0.05).

In terms of occupational status, we found that employed patients had a significantly higher proportion of patients successfully completing on-time CRC screening. Specifically, 79.9% of employed patients were up to date on CRC screening, which was significantly greater than the up-to-date proportion for any other occupational status. The groups with the lowest proportion of patients up to date on CRC screening were unemployed patients (71.6%), disabled patients (66.7%), and full-time students (61%). Additionally, we also found that patients in a relationship had a significantly higher proportion of patients up to date on CRC screening (80.8%) as compared to single patients (73.6%; *p* < 0.05). We also found that patients who were never tobacco smokers had a significantly higher proportion of patients up to date on CRC screening (81.7%) compared to those who were current or past smokers (67%; *p* < 0.05). Finally, the mean BMI of patients who were up to date on cancer screening (29.5 kg/m^2^) was significantly lower than patients who were overdue on CRC screening (30.5 kg/m^2^; *p* < 0.05). This is particularly pertinent as both tobacco smoking and obesity are modifiable risk factors that increase cancer risk.

**Figure 1:**
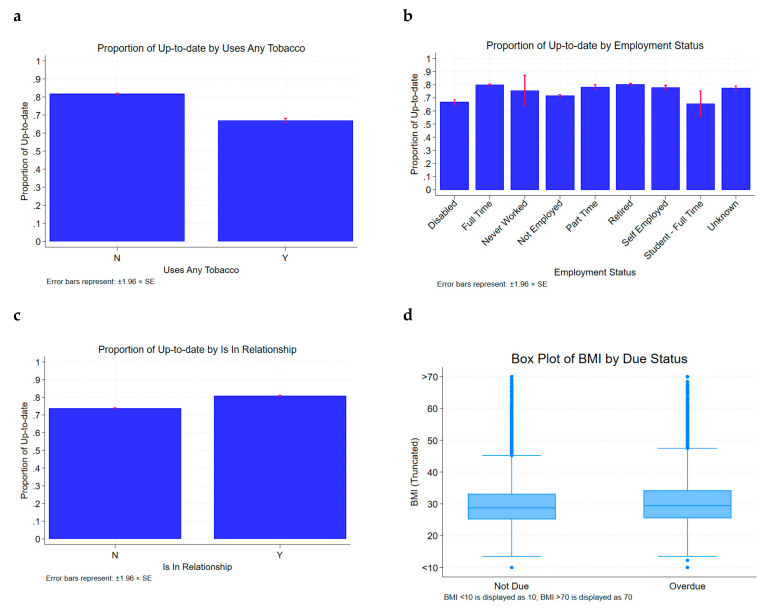
Impact of smoking (**a**), employment status (**b**), relationship status (**c**) and BMI (**d**) on colo-rectal screening completion.

These results are demonstrated in Table 1 and Table 2 and Figure 1. All statistical analysis for this portion was performed using the chi-squared test, apart from the comparison between mean BMIs, which was performed using a two-sample *t*-test.

### 3.2. Analysis of Patients Stratified by Age

As previously discussed, we also stratified our analysis by age to evaluate factors that influenced successful CRC screening in a younger patient population. Overall, we found that patients aged 50 to 55 years had an overall lower proportion of up-to-date patients (77.7%) compared to patients aged 56 to 75 years (78.7%, *p* < 0.05; see Table 3). In addition, among the patients who successfully completed CRC screening on time, we found that patients aged 50 to 55 years had a significantly higher percentage of patients choose Cologuard as their screening mechanism (24.1%) as compared to only 14% of patients aged 56 to 75 years (*p* < 0.05).

Age stratification did reveal some differences in the successful completion of CRC screening in different races. While the trends visualized in the composite patient population persisted for most races even with age stratification, stratifying patients by age did reveal a significantly lower proportion of up-to-date AI/ANs aged 50 to 55 years (69.6%). This was both the lowest among all races in the 50-to-55-year-old age group (*p* < 0.05) and significantly lower than the AI/AN up-to-date proportion of 56-to-75-year-old patients.

Even with stratifying patients by age into the two age groups, the trends noted in the entire, non-stratified patient population for relationship status, occupational status, and smoking status were relatively similar between the two age groups. Specifically, in all three groups (all ages, ages 50 to 55, and ages 56 to 75), patients in a relationship, employed patients, and non-smokers had higher proportions of patients up to date on CRC screening as compared to other categories in those groups. Interestingly, the overall BMI in the younger patient population (30.7) was significantly greater than the overall BMI in the older patient population (29.4; *p* < 0.05). Nevertheless, the trend of up-to-date patients having a significantly lower BMI than overdue patients persisted even with age stratification.

## 4. Discussion

In this study, we have identified patient characteristics, including but not limited to race, that may contribute to disparities in colorectal cancer screening. Additionally, given the recent change in the recommended age of CRC screening initiation to encompass patients aged 45 to 50 years old, we have evaluated the younger patients in our cohort (patients aged 50 to 55 years old) to serve as a surrogate for what may influence successful completion of CRC screening in the younger, 45-to-50-year-old age range in the coming years.

### 4.1. Racial Disparities in CRC Screening

With regard to our patient population as a composite, we identified that patients who identify as Pacific Islanders, East Asian, or White have the highest percentage of up-to-date colorectal cancer screening. In contrast, we have demonstrated that Asian Indians in our cohort of patients consistently have one of the lowest proportions of successful colorectal cancer screening completion. This is an interesting finding, as prior research has demonstrated that East Asians and Pacific Islanders typically have lower screening compliance, particularly as compared to White patients [4,11,12,13]. Notably, much of the research establishing the disparity between East Asians/Pacific Islanders and White patients utilized data from 2015 and prior. The reduction in this disparity that our findings demonstrate could indicate that this past literature brought a previously more marked screening disparity of East Asians/Pacific Islanders to light, resulting in targeted interventions and increased awareness amongst primary care physicians and gastroenterologists, and subsequently yielding a reduction in this disparity for these populations. Indeed, there have been multiple randomized controlled trials that evaluated the efficacy of culturally tailored interventions to increase screening rates among these individuals [4,14,15,16]. Further research is needed to support this notion, as there are certainly other contributing factors that could have impacted the differential screening success rates for these individuals, namely that our study involves a single health system with a patient population that generally has a higher mean income and education level.

Conversely, the persistent disparity that we note for Asian Indians may be related to the connotation that these individuals are at relatively low risk for CRC, with some prior research demonstrating that Asian Indians have the lowest risk of CRC development compared to other Asian ethnicities [17]. It is possible that this perceived low risk for Asian Indian individuals causes physicians and patients to feel a lower urgency for these individuals to be screened, perhaps supported by the fact that younger Asian Indians aged 50 to 55 years have a significantly lower proportion of up-to-date patients than older Asian Indians aged 56 to 75 years (*p* < 0.05) and most other races in this age group. Nevertheless, as modifiable risk factors for CRC, including obesity, type 2 diabetes mellitus, alcohol use, tobacco smoking, and processed food consumption, are on the rise in the Western world, where the Asian Indian population continues to rise, it is important to acknowledge this disparity, as it could lead to a disproportionately elevated mortality in this group in the coming years.

Prior research has demonstrated the need for disaggregation of the Asian American and Pacific Islander (AAPI) community, given the marked heterogeneity in the cultural groups that comprise this group [18]. In fact, prior research has established that with breast cancer, there exist significant variations in the disease burden between subgroups of the AAPI community. While our study focuses on screening, our results demonstrate a similar notion that disaggregating the AAPI group reveals unique trends among the culturally and geographically unique groups that comprise AAPI (East Asians, Asian Indians/South Asians, and Pacific Islanders).

Another interesting trend that we discovered is that while AI/AN had a relatively middle-of-the-road up-to-date screening percentage in our composite patient population (78.7% of patients up to date), the proportion of AI/AN patients aged 50 to 55 years old (only 69.6% of patients up to date) was the lowest in this age range of all races. This is particularly concerning given that AI/AN patients have one of the, if not the, highest mortality burden from CRC [5,19]. This may indicate that targeting younger AI/AN patients is an extremely important opportunity to significantly reduce the overall mortality burden from CRC.

### 4.2. Disparities in Employment Status

Employment status was also shown to be a significant influencer of CRC screening completion, with employed patients having significantly higher proportions of patients up to date on CRC screening in all patient groups. According to data from the U.S. Bureau of Labor Statistics, educational status and monetary income are generally positively associated with employed status [20]. Disabled patients, who are known to be a marginalized population in most healthcare realms, also have among the lowest proportion of patients up to date on CRC screening [21,22]. Thus, targeting patients of lower socioeconomic status, those with disabilities, and patients not employed is essential to maximize appropriate CRC screening.

### 4.3. Disparities in Relationship Status

In all age groups, single patients have been shown to have lower proportions of up-to-date patients than those in a relationship. Relationship status is not a frequently studied parameter contributing to a possible disparity in a health outcome such as cancer screening but could be related to improved social support resulting in a higher likelihood to meet age-appropriate health metrics. Furthermore, most facilities require patients to have a designated person to drive them home after a colonoscopy due to the effects of anesthesia. This may be another circumstance where increasing patient and physician education and utilization of noninvasive screening modalities may reduce disparity.

### 4.4. Disparities in Modifiable Risk Factors

Both tobacco smoking and obesity are established modifiable risk factors that significantly increase all cancer risk, and colorectal cancer is no exception [23,24,25]. It is therefore particularly concerning that both of these risk factors are significantly more common in patients who are not getting appropriately screened for the disease. Some EHRs have piloted strategies that flag patients’ medical charts when they report tobacco smoking or obesity and automatically alert physicians to discuss tobacco cessation or weight loss strategies, respectively. Given our finding that these at-risk populations may be significantly less likely to complete on-time CRC screening despite an elevated risk, a possible quality improvement strategy would be to provide an additional prompt for the consideration of CRC screening when appropriate.

### 4.5. Impact of Age on CRC Screening

In terms of our comparison of younger and older patients, we did note that the overall proportion of younger patients up to date on screening was significantly less than older patients. This may indicate that younger patients are less likely to complete CRC screening on time; if so, this effect is likely to be magnified when including an even younger patient population in the coming years.

An interesting finding is that younger patients were significantly more likely to opt for the noninvasive strategy of Cologuard for their CRC screening modality. It is possible that the COVID pandemic normalized remote patient interactions with the healthcare system [26], perhaps more so for younger, previously healthy patients who have required fewer in-person healthcare experiences compared to older patients. Furthermore, our older patient population included patients over the age of 65 years, the typical age of retirement in the United States. This may indicate that at-home testing is more appealing to younger patients due to the ability to complete it without taking time off of work. All in all, publicizing noninvasive strategies for CRC screening may maximize CRC screening adherence, particularly for a younger patient population.

### 4.6. Comparison to National Average

In 2021, the average percentage of adults ages 50–75 years up to date on CRC screening in the US was 71.2 percent. It is important to note that overall, the percentage of adults that we noted to be up to date on CRC screening was higher than this. Furthermore, while our study did demonstrate a lower up-to-date CRC screening proportion in the 50–55 year age range, the percentage of 50–55 year old patients who were up to date was notably higher than prior studies [27]. Both the overall and younger-patient-specific higher up-to-date screening proportions are possibly results of our patient population being mostly insured; as previously discussed, urgent care visits and post-Emergency Department and hospital follow-up visits were excluded from analysis, and these visits did not always accurately capture patients’ CRC screening status. The inclusion of these visits could be included in the future to encompass more uninsured patients, though this would be at the expense of a lack of assurance of accurate CRC screening status.

### 4.7. Strengths and Weaknesses

Significant strengths of our study include its power, encompassing over 85,000 patients; inclusion of risk factors not traditionally included in potential causes for CRC screening disparities (employment status, relationship status, smoking status, and BMI), and critical evaluation of younger patients, especially given the recent rise in CRC in younger patients. Furthermore, while prior studies evaluating disparities often utilize insurance databases that focus on one or a few insurance subtypes, our analysis included all insured and uninsured patients, though the latter comprised a lower proportion of our patient population. Finally, as previously discussed, our study disaggregated the AAPI group and was subsequently able to reveal unique trends in each subgroup that comprises this larger composite.

Weaknesses of our study include the slight inaccuracy in estimating the characteristics of younger patients only using patients from 50 to 55 years (which was done intentionally to ensure consistency among the compared groups), when new guidelines include those aged 45 to 50 years. Future directions of this study will be to include these younger patients as more data are garnered after the change of guidelines in 2021. Other limitations include the potential for inaccuracy of data in EHRs, temporal delay between screening test completion and the collection of variable information, and the innate possible inaccuracy of self-reported data.

### 4.8. Future Directions

In this study, we have revealed interesting trends in CRC screening, with a focus on younger patients. In the coming years, we would like to expand this analysis to continue to evaluate trends in successful CRC screening completion in younger patients and encompass the 45-to-50-year-old age range. In addition, we are working to evaluate how the studied variables (age, occupation status, etc.) could contribute to successful CRC screening in conjunction.

Finally, a very important future direction that we are currently working towards is the development of culturally relevant and clinically feasible quality improvement initiatives to address the identified disparities in successful CRC screening.

## 5. Conclusions

In our cohort of over 85,000 patients, we have found that certain patient characteristics, namely Asian Indian and American Indian/Native American race, unemployed/disabled occupational status, single relationship status, history of current or prior tobacco smoking, and higher BMIs, are associated with significantly lower proportions of patients up to date on CRC screening. These characteristics are for patients and healthcare professionals to recognize to improve CRC screening rates in these groups, whether by increased awareness or implementation of quality improvement initiatives specifically targeting these groups.

Furthermore, we identified that younger patients are overall less likely to undergo age-appropriate CRC screening, with AI/AN patients having a notably low proportion of up-to-date patients. Younger patients do seem to have a higher proportion of patients opting for noninvasive screening strategies, implying that initiatives publicizing the availability of noninvasive screening modalities may be particularly successful in improving CRC screening rates in younger patients.

## Figures and Tables

**Table 1:** Baseline patient characteristics in age-stratified patient groups.

Patient Characteristics	Age 50–55 N (%)	Age 56+ N (%)
**Race**		
Pacific Islander	40 (0.2%)	68 (0.1%)
East Asian	1328 (7.6%)	2908 (4.7%)
White	10,016 (57.5%)	40,880 (66.2%)
American Indian or Alaskan Native (AI/AN)	46 (0.3%)	165 (0.3%)
Black or African American (B/AA)	5640 (32.4%)	17,080 (27.7%)
Asian Indian	359 (2.1%)	611 (1.0%)
**Gender Identity**		
Female	11204 (58.4%)	37540 (56.9%)
Male	7974 (41.6%)	28466 (43.1%)
	**Insurance Status**	
Insured	18,817 (99.5%)	65,231 (99.7%)
Uninsured	100 (0.5%)	229 (0.3%)
**Occupational Status** *		
Employed (Full time)	14,315 (84.4%)	35,637 (79.3%)
Unemployed	2150 (12.7%)	6677 (14.9%)
Disabled	457 (2.7%)	2350 (5.2%)
Student (Full time)	42 (0.2%)	251 (0.6%)
**Relationship Status**		
In a Relationship	5788 (29.8%)	21,702 (32.9%)
Single	13,643 (70.2%)	44,305 (67.12%)
**Tobacco Smoking Status**		
Never Smokers	15,290 (92.5%)	54,215 (91.6%)
Current or past smokers	1243 (7.5%)	4981 (8.4%)

* For occupational status, the group with the highest up-to-date and the groups with the three lowest up-to-date percentages are shown.

**Table 2:** The impact of various patient characteristics on CRC screening completion among all patient ages.

Patient Characteristics	Up to Date on CRC Screening N (%)	Overdue (Not Up to Date on CRC Screening) N (%)	*p* Value
**Race**			
Pacific Islander	92 (85.2)	16 (14.8)	<0.05
East Asian	3488 (82.3)	748 (17.7)	
White	40,250 (79.1)	10,646 (20.9)	
American Indian or Alaskan Native (AI/AN)	166 (78.7)	223 (23)	
Black or African American (B/AA)	17,523 (77.1)	5197 (22.9)	
Asian Indian	727 (74.9)	243 (25.1)	
**Occupational Status** *			
Employed (Full time)	39,950 (79.9)	10,002 (20.1)	<0.05
Unemployed	6321(71.6)	2506 (28.4)	
Disabled	1872 (66.7)	935 (33.3)	
Student (Full time)	61 (65.6)	32 (34.4)	
**Relationship Status**			
In a Relationship	46,819 (80.8)	11,129 (19.2)	<0.05
Single	20,055 (73.6)	7182 (26.4)	
**Tobacco Smoking Status**			
Never Smokers	56,785 (81.7)	12,720 (18.3)	<0.05
Current or past smokers	4170 (67)	2054 (23)	
**Mean BMI** (kg/m^2^)	29.5 kg/m^2^	30.5 kg/m^2^	<0.05

* For occupational status, the group with the highest up-to-date and the groups with the three lowest up-to-date percentages are shown.

**Table 3:** (**a**) A comparison of the overall successful completion of CRC screening and the screening modality used in patients stratified by age. (**b**) A comparison of race, relationship status, and BMI in patients up to date and overdue on CRC screening, stratified by age.

(a)							
	Age 50–55				Age 56–75		*p* Value
	Up-to-Date N (%)		Overdue N (%)		Up-to-Date N (%)	Overdue N (%)	
**Overall n (%)**	14,910 (77.7)		4268 (22.3)		51,964 (78.7)	14,043 (21.2)	*p* < 0.05
Form of CRC screening chosen *							
**Cologuard**	2588 (24.1)		N/A		7275 (14.0)	N/A	*p* < 0.05
**Colonoscopy**	10,456 (70.1)		N/A		41,255 (79.4)	N/A	*p* < 0.05
**(b)**							
	**Age 50–55**				**Age 56–75**		
	**Up-to-Date** **N (%)**	**Overdue** **N (%)**		***p* Value**	**Up-to-Date** **N (%)**	**Overdue** **N (%)**	***p* Value**
**Race**							
Pacific Islander	35 (87.5)	5 (12.5)		*p* < 0.05	57 (83.8)	11 (18.8)	*p* < 0.05
East Asian	1073 (80.7)	255 (19.2)			2415 (83)	493 (17)	
White	7865 (78.5)	2151 (21.4)			32,385 (79.2)	8495 (20.8)	
AI/AN **	32 (69.6)	14 (30.4)			134 (81.2)	21 (18.8)	
B/AA **	4352 (77.1)	1288 (22.8)			13,171 (77.1)	3909 (22.9)	
Asian Indian	258 (71.9)	101 (28.1)			469 (76.8)	142 (23.2)	
**Employment Status**							
Employed (Full time)	11,334 (79.2)	2981 (20.8)		*p* < 0.05	28,616 (80.3)	7021 (19.7)	*p* < 0.05
Unemployed	1517 (70.6)	633 (29.4)			4804 (71.9)	1873 (28.1)	
Disabled	299 (65.4)	158 (34.6)			1573 (66.9)	777 (33.1)	
Student (Full time)	24 (57.1)	18 (42.9)		*p* > 0.05	237 (72.5)	14 (27.5)	
**Relationship Status**							
Single	4268 (73.7)	1520 (26.3)		*p* < 0.05	16,040 (73.9)	5662 (26.1)	*p* < 0.05
In a relationship	10,895 (79.9)	2748 (20.1%)			35,924 (81.1)	8381 (18.9)	
**Tobacco Smoking Status**							
Never Smokers	12,446 (81.3)	2844 (18.6)		*p* < 0.05	44,339 (81.8)	9876 (18.2)	*p* < 0.05
Current or past smokers	817 (65.7)	426 (32.7)			3353 (67.3)	1628 (32.7)	
**Mean BMI**							
Mean BMI (kg/m^2)^)	30.4	31.7		*p* < 0.05 ***	29.2	30.2	*p* < 0.05 ***

* Only the two screening modalities with the highest use rate are reported, as the number of patients who chose other screening modalities was negligible (n < 100 patients) in comparison to the total population size (n ≈ 85,000). ** AI/AN = American Indian/Alaskan Native. B/AA = Black/African American. *** Statistical analysis for this comparison was performed using a two-sample *t*-test. All other comparisons were performed using a chi-squared analysis.

## Data Availability

All data are available through this manuscript.

## Abbreviations

The following abbreviations are used in this manuscript:

CRC	Colorectal cancer
BMI	Body mass index
AI/AN	American Indian/Alaskan Native
